# An Autonomous Task Assignment Paradigm for Autonomous Robotic In-Space Assembly

**DOI:** 10.3389/frobt.2022.709905

**Published:** 2022-02-25

**Authors:** Joshua Moser, Julia Hoffman, Robert Hildebrand, Erik Komendera

**Affiliations:** ^1^ Field and Space Experimental Robotics Lab, Mechanical Engineering, Virginia Tech, Blacksburg, VA, United States; ^2^ Grado Department of Industrial and Systems Engineering, Virginia Tech, Blacksburg, VA, United States

**Keywords:** autonomous robotic assembly, in-space assembly, mixed integer programming, markov decision process, task allocation, task sequencing, stochastic assembly environment

## Abstract

The development of autonomous robotic systems is a key component in the expansion of space exploration and the development of infrastructures for in-space applications. An important capability for these robotic systems is the ability to maintain and repair structures in the absence of human input by autonomously generating valid task sequences and task to robot allocations. To this end, a novel stochastic problem formulation paired with a mixed integer programming assembly schedule generator has been developed to articulate the elements, constraints, and state of an assembly project and solve for an optimal assembly schedule. The developed formulations were tested with a set of hardware experiments that included generating an optimal schedule for an assembly and rescheduling during an assembly to plan a repair. This formulation and validation work provides a path forward for future research in the development of an autonomous system capable of building and maintaining in-space infrastructures.

## 1 Introduction

As the exploration of space continues to develop, many opportunities arise to advance the current understanding of celestial objects, develop and deploy new experiments, and harness natural resources that have thus far been inaccessible. From building telescopes and space structures too large to launch from Earth [Belvin et al. (2016); Karumanchi et al. (2018); Roa et al. (2020)] to building structures for human habitation on Mars or the Lunar surface [Thangavelautham et al. (2020)], the use of robotic workforces will be instrumental in achieving the next steps in space exploration. These applications will require robotic systems capable of constructing, maintaining, and repairing infrastructures in conditions with limited input from humans. This in-space autonomous assembly problem (ISAAP) can be categorized into three general groups. The sensor network: a complex autonomy system that allows for the robots to observe their surroundings. A task allocation system: a system that will sequence and allocate tasks to complete the assembly. And finally, the local robot autonomy that allows an individual robot to carry out its tasks (this includes the kinematic models, dynamics models, and control logic). The work presented here will focus on the task allocation and assignment portion of this problem since it is relatively unaddressed in the in-space domain. The current state-of-the-art for in-space robot assembly operations are primarily done through teleoperation Rognant et al. (2019). In the few cases where there is autonomy, it is limited and requires human supervision and interaction Lee et al. (2016); Belvin et al. (2016). This paradigm of requiring teleoperation or even human supervised autonomy is not viable for longer distance missions Saunders et al. (2017) nor for missions with increased complexity Rognant et al. (2019). Because this is a newer frontier for autonomous robotics, the literature is rather sparse and there remains much to be explored and developed Rodriguez et al. (2021). As such, there is currently no clearly preferred architecture for this application Nanjangud et al. (2019). Some research for autonomous assembly has been done for particular assembly scenarios such as Ikea furniture assembly Knepper et al. (2013), manufacturing cell Arai et al. (2013), and construction Hartmann et al. (2021). However, the formulations utilized in these applications deal with simple assemblies, predetermined sequences, brute force computation, or combined task allocation and robot motion planning which does not scale well Hartmann et al. (2021). As a result, many of the assembly applications for in-space are not able to be fully autonomous and use manually defined task sequences Karumanchi et al. (2018). A very recent work by Rodriguez et al. (2021) begins to address this with a proposed system that uses a state space that tracks the state of each module in a global context and utilizes a planning architecture that uses graph search algorithms to evaluate proposed assembly sequences.

In this current state, the task scheduling knowledge base is still missing important elements necessary for robotic assembly in the absence of human input. Once such element is the modeling of robotic ability with chance of failure and how that impacts state transitions. A second element is a state formulation that can accommodate state changes reflecting repair operations or the undoing of assembly steps. Finally, continuity constraints (one robot required for the same action across jobs) are another important factor in realistic assembles that have not been modeled in the task scheduling literature. The novel work presented in this paper moves the current state of literature forward and offers a problem formulation capable of articulating robotic ability and how the chances of failure impact state transitions. The formulation also naturally expands to accommodate all possible state transitions that could occur in an assembly, including transitions related to repair or partial disassembly. This work then presents a task sequencing and task to robot allocation solution generation method (assembly schedule generator) that will utilize information encoded in the novel assembly problem formulation and produce assembly schedules in an optimal or near optimal way. This solution generation includes the traditional constraints such as sequence and robot processing time considerations and novel continuity and local workspace considerations. These contributions take the literature a step closer to functional autonomous robotic systems in-space that are capable of replanning and reallocating tasks without the need for human supervision, a necessary capability if autonomous robotic systems are going to assemble and repair structures in an environment with uncertainty without human input.

## 2 Materials and Methods

The contributing factors to the ISAAP can be thought of in three general groups: *assembling agents*, *tasks*, and *environment*. The *assembling agents*, in the context of this work, are robotic units that make up the workforce to complete the assembly. These robotic units often have different levels of ability for completing a range of operations present in an assembly environment. Contributing elements to this variation include mobility, local workspace (reach), and end effector type. In addition to these variations, there is often a stochastic element to each robot’s ability sourced from uncertainty present in the sensor feedback systems, state estimation through a metrology system, the characteristics of the control algorithms, and variations in the environment. To effectively assign robots in complex assembly scenarios, these variations in ability needs to be articulated and accounted for in the assignment process. The second group, *tasks*, includes all of the steps necessary to build an assembly. To accomplish a successful assembly, there are often specific task sequences that must be followed to achieve a viable structure. There may also be a range of connection methods and special tools that are necessary in completing an assembly. A problem formulation must be able to articulate these constraints in a form that a schedule generator can process. The final group, *environment*, also includes important considerations related to the configuration of an assembly problem. Locations for parts and designations for where assembly steps must take place must be included in the formulation to allow the schedule generation algorithm to include spatial considerations in the task sequencing and robot to task allocation. All of these factors highlight the complexity of the ISAAP and underscores the need for a flexible and expansive framework to successfully articulate the different features present in assembly projects.

### 2.1 Stochastic Assembly Problem Definition

To address the features described in the ISAAP, a stochastic assembly problem definition (SAPD) has been develop to provide a formulation that represents the details of the *agents*, *tasks*, and *environment*, casting them into a framework suitable for task allocation. This takes the form of three main groups: *Elements*, the physical and functional characteristics; *Constraints*, to articulate a valid assembly sequence; and *State Representation*, a flexible mathematical model to represent the overall state of the assembly in the context of a stochastic paradigm.

#### 2.1.1 Elements

The Elements portion of the problem formulation describes the “physical” portions of assembly agents, tasks, and environment features in the ISAAP. This is done through four different classes: *Points*, the important locations in the assembly space, *Components*, the structural parts in the assembly, *Joints*, the structural connection information, and *Robots*, the autonomous operators forming the workforce in the assembly problem. Each of these types will contain a property category that will be used as a bookkeeping/information storage area containing the information that *does not* contribute to the current state of the assembly. This can include information such as the element’s name, type, starting location at the beginning of the assembly, etc. The classes that contain the information that *does* affect the state of the assembly will have a second category, states. These stateful information entries will include information such as the current location of the element at a specific time in the assembly, if the element is in position or not, if it is damaged, etc. The following sections expand on the formulation structure for each of these element classes.

##### 2.1.1.1 Points

This class is primarily used to describe locations of interest through the assembly environment. Denoted by the set ***W*** = {*W*_1_, *…* , *W*_*i*_, *… W*_| ***W*** |_}, each point can be thought of as a description of a specific location, position, or position and orientation. Since these designations do not typically change as the assembly progresses, this class will be stateless in most cases. Each point will contain at least two property features: type and location. The type feature allows for different categories of points to be distinguished between and in most cases, at least a reference type will be present to note the different locations. The location feature provides the coordinate of the point in the workspace. The overall formulation does not restrict the type of coordinate frame (Cartesian, cylindrical, etc.) or specifically require orientation information to be included, leaving it open to what fits the assembly problem environment and application the best. Additional property information can be added if required in the context of the assembly.

##### 2.1.1.2 Components

A component, represented in the set ***C*** = {*C*_1_, *…* , *C*_*i*_, *… C*_| ***C*** |_}, is any physical part that is included in the autonomous assembly problem and is not an autonomous operator. This set contains all of the elements that will need to be manipulated for the assembly. Like the points class, each *C*_*i*_ has a set of properties describing the type of component and other information that may pertain to the assembly. The components directly affect the state of the assembly. Therefore, each component is stateful and will contain a set of state information features. There are generally three different state features: in position/not in position, broken/not broken, and current location. The first state, in position/not in position, reflects if the component is in the correct installation location. If it is not, additional actions will be required to move it into position. The broken/not broken state describes if a component is going to need to be replaced or if it can remain. These two states allow for the description of a condition where a component might be in place but installed wrong or when a component has been broken after installation and needs to be removed. Either case of being in the broken state will require a task or task set to be inserted into the assembly sequence to correct the problematic component. Finally, the current location state provides the location information as the component transitions around the environment. The property features for each component, similar to those in points, will contain information such as component type, locations, and weight (if applicable). The location property feature can contain points that are important to the component. Two primary examples are the start location at the beginning of the assembly and the goal location. An additional example includes points on the component necessary for joining. By including these locations in the component definition, their positions and orientations can be with respect to the component’s location rather than the global reference frame. This removes the need for them to be in the state feature category and thereby reduces complexity in the overall assembly state representation.

##### 2.1.1.3 Joints

A joint, represented in the set ***K*** = {*K*_1_, *…* , *K*_*i*_, *… K*_| ***K*** |_}, can be thought of as an element of the assembly problem that is physical but is not in the form of a component or a location. It represents the connection between components in an assembly. This will take the form of welds, bolt joints, etc. If needed, and with a slight abuse of some terminology, this can also be used to describe painting or spreading a sealant on components as well. Similar to the component type, this element contains states and properties in its definition. For a joint, joined/not joined or completion percent of being joined are the two most common state features. If there is a process to completing a joining method, the percentage state representation may be necessary. Alternatively, if the connection is something like a snap connection, having a simple joined/not joined state representation can be sufficient to describe the state. Type, locations, and component list are all examples of the property features that may be included in the joint definition.

##### 2.1.1.4 Robots

The robot element class is the final of the four and represents the autonomous operators used to complete the assembly project. A given robot, represented in the set ***R*** = {*R*_1_, *…* , *R*_*i*_, *… R*_| ***R*** |_}, will have the following state features: idle/busy, current location, current task, and energy level (if applicable). The idle/busy state is used to denote if a robot is available for assignment. The current location, as implied, gives the updating location of the robot and current task represents what the robot is currently working on. This state does not remove the need for the idle/busy state since there are many applications where a robot may not be tasked but is still not available for a task allocation. Energy level, if the information is applicable, can be thought of as a projection of how much longer the robot can operate before it needs to be retired for charging or replacement. In addition to the states, each robot will generally have five property features: robot type, locations, mobility, workspace, and abilities. Mobility and workspace, when present, provide enough information to articulate the reach of a robot (the local workspace) without moving the base, and if a robot is mobile, that is, if the origin of the local workspace can be moved to a new location in the environment. The combination of these two properties allows for a schedule generator to determine if a task is reachable by a specific robot. To define the abilities feature, a set of operation types in the project needs to be defined. These are based on the types of components, joints, and robots in the assembly project and represents the discretized contribution of a robot in a given task. These operations, defined as the set ***O*** = {*O*_1_, *…* , *O*_*i*_, *… O*_| ***O*** |_}, will be described in more depth in section 2.1.2.1. For now, it is enough to say that each robot will have an entry for each *O*_*i*_ ∈ ***O*** in its abilities property describing its capability or limitations in completing that operation. In its simplest form, this can be thought of as a processing time, describing how long the robot will take to complete the operation. In higher fidelity formulations this entry will include stochastic information in the form of distributions. The processing time, approximated as a normal distribution, will describe the variation present in completing the specific operation by the robot. This can include variation introduced from a need to adjust the robot’s grip on a component or correcting for an overshot location. Additionally, probabilistic information for minor and major failures can also be included at this point. Minor failures, defined as failures in completing an operation that do not break the component or portions of the assembly, can be separated out of the processing time and modeled as a separate normal distribution paired with a geometric distribution to model the number of times the robot might need to attempt aligning a component and how long each occurrence may take. The chance of a major failure, one that damages other elements in the assembly triggering a need for new tasks to be inserted to undo, replace, or repair the damaged components can also be modeled with a Bernoulli distribution. All of these distributions can be replaced to fit the characteristics for the specific robots and scenarios in a given assembly without changing the architecture of the SAPD.

#### 2.1.2 Constraints

The constraints portion of the SAPD frames the requirements describing the criteria for a valid assembly. This will includes a job shop type formulation to discretize and articulate the steps needed to complete an assembly, precedence constraints to ensure tasks are completed in the required order, continuity constraints and machine validity constraints to ensure that the correct machines are used on tasks, and finally, distance constraints to encapsulate the impact of robots traveling between tasks in the assembly. A preliminary introduction to some of these constraints was published in Moser et al. (2020), however the follow sections provide additional details and constraints necessary to frame the full assembly problem.

##### 2.1.2.1 Job Shop Scheduling Problem Formulation

Variants of the job shop scheduling problem (JSSP) formulation have been used throughout literature as a way to describe a project in terms of the tasks that need to be completed, represented as jobs, and the individual machine contributions in each of those tasks, represented by operations (Ozgüven et al., 2010; Chaudhry and Khan (2016)). In this paradigm, the jobs, represented by the set ***J*** = {*J*_1_, *…* , *J*_*i*_, *… J*_| ***J*** |_}, make up the tasks that must to be assigned and processed to complete an assembly problem. Each type of job will have a set of operations (***O***_***j***_) as sub-elements representing different robot contributions in completing a job. Therefore, if a job can be completed by one robot alone, it will contain one operation. However, if a method of completing a job requires two robots, that will be represented by two different operations. In some cases, there may be more than one way to complete a job. This is represented by a set of process plans (***P***_***j***_) where each process plan is a set of operations describing how job *j* can be completed.

##### 2.1.2.2 Precedence

Many assemblies require specific task sequences to successfully reach the completed state. This is reflected in scenarios such as: a component can not be connected until it has been moved into position. To represent this constraint set, a Directed Acyclic Graph (DAG), *G*_*p*_ (*V*_*p*_, *α*_*p*_), is used to embed the precedence constraints. Each vertex, *V*_*p*_, represents a job in the assembly project and each arc, *α*_*p*_, represents a precedence constraint, thereby encoding the project precedence constraints in the structure of the graph. Representing precedence this way allows for ordering constraints to be described only in terms of the jobs the must immediately proceed or follow it to provide a sequence framework that the overall assembly must follow to reach a valid completion state.

##### 2.1.2.3 Continuity

In an assembly project, there may be occasions where a robot needs to continue the same operation between two jobs. An example of this is illustrated by a robot tasked with aligning a part before it is fixed into position. The same robot must also be tasked with holding that part during the actual affixing job since the use of a different robot would result in the loss of alignment. By using the precedence DAG to require the two jobs to come in direct sequence and the continuity constraint requiring the robot to be constant across the two jobs, the resulting assignment will reflect the requirement of using the same robot for the two tasks without being assigned something in-between. For this formulation, the continuity constraint, represented by the set ***H*** = {(*arcs*, *O*)}, is framed as arcs from the precedence DAG and operation type parings. The arcs describe which jobs these constraints apply to and the operation type represents which operations needs to be assigned to the same robot across the arcs.

##### 2.1.2.4 Valid Robot Section

In an assembly problem, there are instances in which a specific robot must be used for a job even if other robots are technically capable of completing it. This constraint can be used to force certain robots to work on a specific job. It can also be used to reduce the number of job types. For example, if the robots each have a specific job reflecting how they are stored at the end of an assembly, only one storage job type needs to be defined. This constraint can then be used to require individual robots to complete the storage job that is applicable to them. To formulate this constraint, a set of pairs, ***V*** = {(*J*, *R*)}, is defined that represents what robots can complete what jobs.

##### 2.1.2.5 Distance

Finally, spatial information, part of the *environment* consideration, must be included for many assembly problems. This consideration is modeled using a fully connected graph (FCG), *G*_*d*_ (*V*_*d*_, *E*_*d*_). There are two different ways that this constraint can be implemented. The vertices, *V*_*d*_, can either represent specific jobs or the locations of points in the assembly space. In both cases the edges, *E*_*d*_, represent the distances between the features modeled by the vertices. Depending on what other autonomous capabilities are present or what additional information is known about the environment, these edge values can represent direct point to point distance, such as a Cartesian distance, or they can represent the distance of a path that must be taken to move between the vertices. In either case, the functionality of the FCG remains the same and provides information to the schedule generator allowing it to determine the time commitment and requirement of moving between specific jobs or locations for a given robot.

#### 2.1.3 State Representation

The definitions above provide a description method for each element in the assembly project and their individual states when applicable. The aggregated states of all these elements form the basis for a description of the overall state of the assembly. For the purpose of task assignment, it is necessary to model how the state of an assembly will change for a particular robot to task (job/operation) assignment. As noted earlier, a realistic autonomous assembly problem will often contain stochastic elements that factor into the state transition of the assembly. To accommodate this, the SAPD models the autonomous assembly as a Markov Decision Process (MDP), represented as the tuple (***S***, ***A***, *P* (*s*′|*s*, *a*), *R* (*s*′, *s*)), containing the state set, action set, transition probability, and transition reward respectively.

##### 2.1.3.1 State Space

In the MDP formulation, ***S*** is the set of all possible states the assembly can take, ***S*** = {*S*_*c*_, *S*_*J*_, *S*_*R*_}. Each unique state, *s* ∈ ***S***, is a different combination of the stateful elements present in the assembly. Many of these are naturally discretized as in the case of a component being in position or not. Other states, such as location and percent completion, must be approximated by a discretized unit. The level of discretization will be a function of the schedule generator’s sensitivity to state space size. Regardless of the level of discretization, the following formulation is still valid.

##### 2.1.3.2 Action Space

The action space, ***A***, is the space representing all of the possible actions in the assembly problem. These actions take the form of assigning different robots to different job/operation pairs: *a*: *r* → (*j*, *p*, *o*) where *r* ⊆ ***R***, *j* ⊆ ***J***, *p* ∈ ***P***_***j***_, *o* ⊆ ***O***_***jp***_. In an autonomous assembly scenario, the schedule generator will assign these actions during the task allocation process.

##### 2.1.3.3 Transition Probability

**FIGURE 1:**
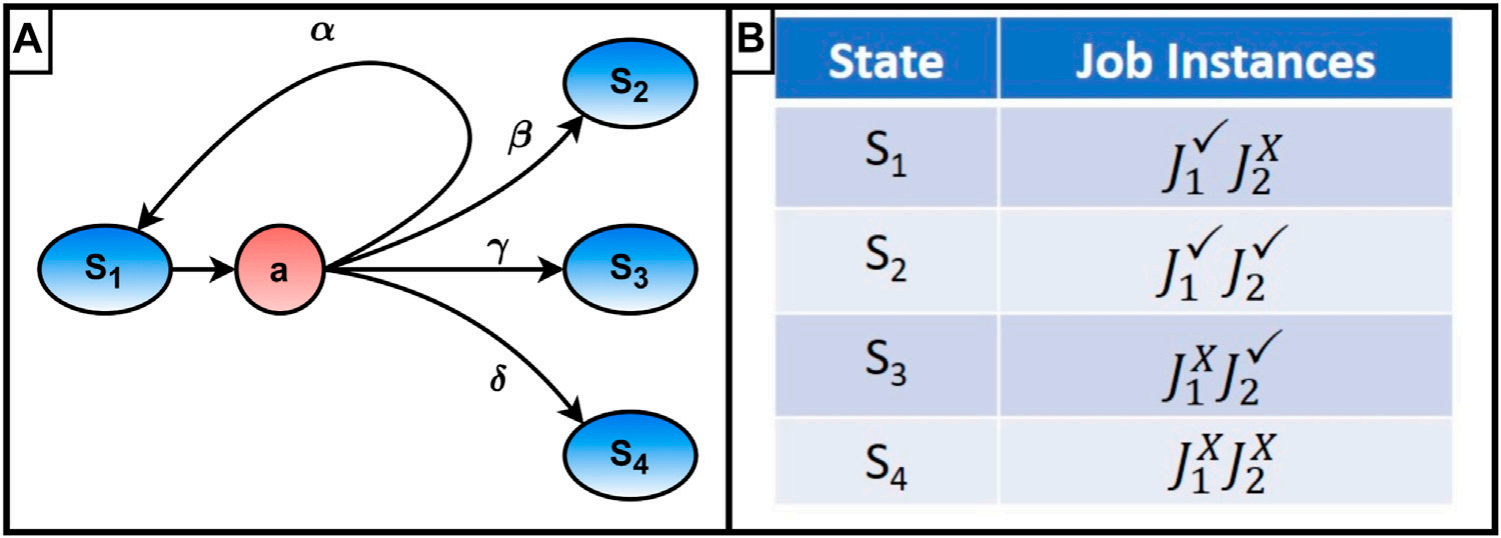
**(A)** Example Markov decision process state transition graph, **(B)** States in example Markov decision process.

The transition probabilities, *P* (*s*′|*s*, *a*), can be thought of as a measurement of how likely a certain state transition is (*s* → *s*′) given an action assignment (*a*). These transition probabilities are a key feature of this problem formulation, allowing it to encapsulate much of the stochastic nature present in the assembly problem. These probabilities are sourced from the abilities in the SAPD formulation for the robot class described earlier. The model functionality is demonstrated in the simple MDP graph in Figure 1 panel A where a simple assembly requires a block to be moved into position and connected to the ground. Letting *J*_1_ represent moving the block and *J*_2_ connecting it in place, state *s*_1_ represents the state where the block has been moved into position but has yet to be connected. The other three states are shown in Figure 1 panel B and represent the block fastened in the correct location (*s*_2_), fastened in the incorrect location (*s*_3_), and finally, not fastened and not in the correct location (*s*_4_). Based on the action assignment *a*, a robot (*R*1) is assigned to connect the block into position. If there is a 25% chance of the robot failing to complete the connection task $\left(P_{R_{1}}\left(J_{2}^{\times }\right)=0.25\right)$ , a 50% chance that the failure will dislodge the block from its correct location $P_{R_{1}}\left(J_{1}^{\times }|J_{2}^{\times }\right)=0.50\left.\right)$ , and a 0.1% chance a successful connection will dislodge the block $\left(P_{R_{1}}\left(J_{1}^{\times }|J_{2}^{✓}\right)=0.001\right)$ , the transition probabilities of ending up in the four different states are:

$$\begin{array}{cc} P_{a}\left(\alpha \right) & =P_{R_{1}}\left(J_{1}^{✓}|J_{2}^{\times }\right)P_{R_{1}}\left(J_{2}^{\times }\right)=0.125\\ P_{a}\left(\beta \right) & =P_{R_{1}}\left(J_{1}^{✓}|J_{2}^{✓}\right)P_{R_{1}}\left(J_{2}^{✓}\right)=0.7425\\ P_{a}\left(\gamma \right) & =P_{R_{1}}\left(J_{1}^{\times }|J_{2}^{✓}\right)P_{R_{1}}\left(J_{2}^{✓}\right)=0.0075\\ P_{a}\left(\delta \right) & =P_{R_{1}}\left(J_{1}^{\times }|J_{2}^{\times }\right)P_{R_{1}}\left(J_{2}^{\times }\right)=0.125 \end{array}$$ (1)

By structuring the state representation with this MDP structure, the possibility of *failures*, *restarts*, and a need for *backtracking* to repair in an assembly project can be quantified and taken into account by the autonomy. This form also allows for impossible paths to be blocked by asserting zero probability for the impossible transitions. In this general form, *job insertions* are not an issue since they would simply represent a different action option at states. Unknown states would also not be a concern since this type of formulation describes **every possible state** by design. This is an important element when it comes to replanning since the required transitions for replanning are *already embedded* in this formulation. It should be acknowledged here that while this is possible in the theoretical case, in practice, extremely large state spaces are not practical for many schedule generator methods to handle. Therefore, some schedule generation formulations will use a reduced set of states in the MDP model to approximate the entire assembly. However, the formulation itself is capable of scaling as required for the specific assembly problem and schedule generation method being utilized.

##### 2.1.3.4 Transition Reward

The final element of the MDP formulation is the state transition reward, *R*_*a*_ (*s*′, *s*). This represents how beneficial a state transition (*s* → *s*′) is with respect to completing the assembly. This might take the form of the inverse cost of transition, a value rewarding specific job completions, a measure of how close the new state is to project completion, or focusing on elements such as penalizing the entering of broken states (states where something in the assembly is broken). The general formulation does not constrain how this reward is generated, leaving a framework in place to be utilized by the different schedule generation methods. Some examples of the reward might be a time cost, or some penalty value utilized in a schedule generator’s solver.

### 2.2 Mixed Integer Programming Formulation

Now that a framework has been developed to describe the ISAAP, different solution methodologies can be developed and implemented to produce assignment schedules to describe task sequences and robot to task allocations that lead to a valid assembled state. These solutions can take many different forms depending on the objective behind the schedule, the capability of the scheduling algorithm, and the specifics of a given ISAAP problem being modeled. In this work, a mixed integer programming (MIP) methodology will be presented to generate optimal schedules to the ISAAP. A benefit to using a MIP formulation is the ability to quantify how close a generated schedule is to an optimal schedule. The branch and bound solving method naturally bounds the optimal solution between the current best integer solution and the linearly relaxed solution. Using these bounds, and optimal solution can be verified or some measure of how far a solution is from optimal can be quantified. Current mixed integer programming formulations for robot task assignment include considerations for distance (Booth et al. (2016)), job sequence considerations (Gini (2017)), and cross robot dependencies (Korsah et al. (2012)). The formulation presented below expands the concepts in these formulation to include considerations for robotic task assignment by framing the constraint set to account for sequence dependent operation requirements and local workspace considerations with respect to the robots.

#### 2.2.1 Formulation Elements

The developed MIP formulation can be thought of in three main sections: *variables*, *objective function*, and *constraints*. The *variables* will describe the binary and continuous elements being modified by the MIP solver as it seeks, in this case, to minimize the *objective function* within the limitations of what makes a valid assembly sequence and task allocation described by the *constraints*. First, a mathematical representations of the SAPD elements must be mapped for use in the MIP formulation.

##### 2.2.1.1 Sets

The sets of robots, jobs, process plans, and operations sets from the SAPD are all used directly in the MIP formulation. Additionally, a set of arcs from the precedence DAG must be defined to include the precedence information into the MIP formulation domain. Using these arcs, the continuity constraint information is also included. In the MIP formulation, all of these sets are defined as:

$$\begin{array}{llll} & R & & \;\text{Set of all robots}\\ & J & & \;\text{Set of all jobs}\\ & P_{j} & & \;\text{Set of all process plans for job}\;j\\ & O_{jp} & & \;\text{Set of all operations for a given process plan}\;P_{j}\\ & A & & \;\text{Set of all precedence arcs}\\ & O_{a} & & \;\text{Set of all operations that require continuity for a given}\;a\in A \end{array}$$

##### 2.2.1.2 Parameters

In addition to the sets, several parameters (values that will not change within the model during solving) must be defined. The first is the processing time, a representative value pulled from the robot ability information. This value is used by the scheduler to model how long it will take a given robot to complete a certain operation. In addition to the process time, a time for traversing distance must be defined. This value, noted as the setup time since it is the time a given robot will take to move from one job to another, is derived from the distance between two jobs, pulled from the FCG in the SAPD and the given robot’s ability to move around the environment. Additionally, parameters describing if a robot is mobile, if a job begins within a robot’s local workspace, and if a specific job can be allocated to a specific robot are also defined from the SAPD. Finally, a very large constant number must be established for use in the MIP constraint definition to allow for certain constraints to be “turned off” under certain conditions. This will be explained further in section 2.2.1.5. The definition of these parameters take the form:

$$\begin{array}{llll} & T_{jpor} & & \text{The processing time of operation}\;o\;\text{in the process plan}\;p\;\text{for job}\;j\;\text{by robot}\;r\\ & S_{jj^{\prime }r} & & \text{Setup time of robot}\;r\;\text{moving from job}\;j\;\text{to job}j^{\prime }\\ & m_{r} & & 1\text{ if robot}\;r\;\text{is mobile and 0 if it is not}\\ & g_{jr} & & 1\text{ if job}\;j\;\text{is within the local workspace}\left(\text{grasping range}\right)\text{of robot}\;r\\ & v_{jr} & & 1\text{ if robot}\;r\;\text{can work on job}\;j\;\text{and a}\;0\;\text{if it can not}\\ & L & & \text{A very large number used to help toggle constraint equations} \end{array}$$

##### 2.2.1.3 Variables

###### Binary

The binary variables represent decisions. These can be thought of as a 1 or 0 switch, a 1 if the decision was a yes and a 0 if the decision was a no. These decisions include if a robot is assigned to a specific job, if a specific process plan is chosen for a given job, and what operation a robot is assigned to for a given job, process plan, and operation configuration. They also include what job a robot starts working on, what jobs it transitions between, and what job it ends on. The definition for each of these binary variables take the form:

$$\begin{array}{llll} & x_{jr} & & 1\text{ if job}\;j\;\text{ was assigned to robot }\;r,\text{ 0 otherwise}\\ & x_{jp} & & 1\text{ if process plan }\;p\;\text{ is chosen for job }\;j,\text{ 0 otherwise}\\ & x_{jpor} & & 1\text{ if robot }\;r\;\text{ is assigned to operation }\;o\;\text{ in process plan }\;p\;\text{ for job }\;j,\text{ 0 otherwise}\\ & x_{jr}^{start} & & 1\text{ if robot }\;r\;\text{ starts project with job }\;j,\text{ 0 otherwise}\\ & x_{jj^{\prime }r} & & 1\text{ if robot }\;r\;\text{ moves from job }\;j\;\text{ to }\;j^{\prime },\text{ 0 otherwise}\\ & x_{jr}^{end} & & 1\text{ if job }\;j\;\text{ is the last job that robot }\;r\;\text{ works on in the project, 0 otherwise} \end{array}$$

###### Continuous

In addition to the binary variables, there is a set of continuous variables used to note important time quantities that the model must set. These include when a job is started, when it is completed, when a specific operation is completed within a job, and when a robot completes its work on a job. Additionally, the times when a robot starts traveling between two jobs and finishes traveling between two jobs are also modeled. Finally, the overall project duration (the project makespan in job shop terms) is defined. In this formulation, these take the form:

sjThe start time of jobjcjThe completion time of jobjcjpoThe completion time of operation o in process planp for jobjcjrThe time robot r completes jobjsjj′rThe start time for robot r moving from jobj to jobj′ forj≠j′cjj′rThe completion time for robot r moving from jobj to jobj′ for j≠j′cmaxThe upper bound on completion timethe project makespan

##### 2.2.1.4 Objective

For this formulation the objective is to minimize the overall time it takes to complete the assembly project. This will drive the model to minimize the amount of time a robot is idle and it will encourage the solver to choose robot assignments that pick the best robot for a given job/operation while taking into account how long it will take the robot to move to the job it has been assigned. This is defined as:

$$\min\;c_{\max}$$ (2)

##### 2.2.1.5 Constraints

To enforce all of the constraints present in the problem formulation a series of linear constraints must be defined. These can be broken into five different groups: Assignment constraints, continuity constraints, robot flow constraints, mobility and workspace constraints, and finally, timing constraints.

###### Assignment Constraints

In the first set of constraints, the fact that a job can only be performed one way across the whole assembly is enforced. This means only one process plan can be used per job in the assembly (Eq. 3). When a robot is assigned to a job it must also be assigned to one and only one operation in that job (Eq. 4) and (Eq. 5), which must be included in the list of operations in the process plan the scheduler selected (Eq. 6) and (Eq. 7). If the robot is not assigned to a job it can not work on any operations in that job (Eq. 8). These principles are enforced by the following constraints:

$$\underset{p\in P_{j}}{\sum }x_{jp}=1\forall j\in J$$ (3)

$$\underset{p\in P_{j}}{\sum }\underset{o\in O_{jp}}{\sum }x_{jpor}\leq 1\forall j\in J,r\in R$$ (4)

$$x_{jp}=\underset{r\in R}{\sum }x_{jpor}\forall j\in J,p\in P_{j},o\in O_{jp}$$ (5)

$$x_{jpor}\leq x_{jp}\forall j\in J,p\in P_{j},o\in O_{jp},r\in R$$ (6)

$$x_{jpor}\leq x_{jr}\forall j\in J,p\in P_{j},o\in O_{j}p,r\in R$$ (7)

$$x_{jr}=\underset{p\in P_{j}}{\sum }\underset{o\in O_{jp}}{\sum }x_{jpor}\forall j\in J,r\in R$$ (8)

###### Continuity Constraint

If there is a continuity requirement, that is, if the same robot must work the same operation between two jobs, that is enforced with the constraint:

$$x_{j^{\prime }p^{\prime }or}\leq x_{jpor}\;\forall p^{\prime }\in P_{j^{\prime }},p\in P_{j},o\in O_{\left(j^{\prime },j\right)},\left(j^{\prime },j\right)\in A,r\in R$$ (9)

###### Robot Workflow Constraints

In addition to the assignment considerations above, the manner in which robots work on jobs must also be enforced. In a robot’s workflow, there must only be one start and one end node (Eq. 10) and (Eq. 11). A robot must only start and stop working on a given job once in its workflow before moving on to the next job (Eq. 12) and (Eq. 13), and it must not loop back to the same job after completing it (Eq. 14):

$$\underset{j\in J}{\sum }x_{jr}^{start}=1\forall r\in R$$ (10)

$$\underset{j\in J}{\sum }x_{jr}^{end}=1\forall r\in R$$ (11)

$$x_{j^{\prime }r}=x_{j^{\prime }r}^{start}+\underset{j\in J}{\sum }x_{jj^{\prime }r}\forall j^{\prime }\in J,r\in R$$ (12)

$$x_{jr}=x_{jr}^{end}+\underset{j^{\prime }\in J}{\sum }x_{jj^{\prime }r}\forall j\in J,r\in R$$ (13)

$$x_{jjr}=0\forall j\in J,r\in R$$ (14)

###### Mobility and Workspace Constraints

A robot cannot be assigned to a job unless the robot is a valid machine for that job (Eq. 15). It must also have access to the job, either having it within its local reach or having the ability to move around the assembly environment (Eq. 16):

$$x_{jr}\leq v_{jr}\forall j\in J,r\in R$$ (15)

$$x_{jr}\leq g_{jr}+m_{r}\forall j\in J,r\in R$$ (16)

###### Timing Constraints

Finally, timing constraints are necessary to ensure that the jobs are started and completed in a valid manner adhering to the limitations for the assembly. Jobs can not be completed until after all of the operations in the job are completed (Eq. 17) and the completion time of a job must equal the start time plus the time it takes to process the job (based on the assigned robots) (Eq. 18). The start time of a job can not be less than the completion time of the previous job worked on by that robot plus the amount of time it took the robot to get to the current job (Eq. 19). Additionally, a job can not be started until all the jobs prior to it in the precedence DAG are completed (Eq. 20). A robot can not finish working on a job until the job is completed (Eq. 21), the time a robot starts moving to a new job can not be before it finishes the job it is working on (Eq. 22), and it can not complete the travel time until the amount of time to move between jobs has passed (Eq. 23). Finally, the overall project completion time (the makespan) can not be less than the largest job completion time (Eq. 24). As noted in section 2.2.1.2, a very large constant (*L*) can be used to turn off constraints when applicable. This is common practice for MIP models since it shifts the value of one side of the constraint based on the configuration of a binary variable. The two constraints using this technique along with the other timing constraints take the form:

$$c_{j}\geq c_{jpo}\forall j\in J,p\in P_{j},o\in O_{jp}$$ (17)

$$c_{j}\geq s_{j}+\left(T_{jpor}\right)x_{jpor}\forall j\in J,p\in P_{j},o\in O_{jp},r\in R$$ (18)

$$s_{j^{\prime }}\geq c_{j}+S_{jj^{\prime }r}x_{jj^{\prime }r}-L\left(1-x_{jj^{\prime }r}\right)\forall j\in J,j^{\prime }\in J\backslash \left\{j\right\},r\in R$$ (19)

$$s_{j^{\prime }}\geq c_{j}\forall \left(j^{\prime },j\right)\in A$$ (20)

$$c_{jr}=c_{j}\forall j\in J,r\in R$$ (21)

$$s_{jj^{\prime }r}=c_{j}-L\left(1-x_{jj^{\prime }r}\right)\forall j\in J,j^{\prime }\in J\backslash \left\{j\right\},r\in R$$ (22)

$$c_{jj^{\prime }r}=c_{j}+S_{jj^{\prime }r}x_{jj^{\prime }r}-L\left(1-x_{jj^{\prime }r}\right)\forall j\in J,j^{\prime }\in J\backslash \left\{j\right\},r\in R$$ (23)

$$c_{\max}\geq c_{j}\forall j\in J$$ (24)

## 3 Experiment

As a validation for the SAPD and the MIP schedule generation method, a multi-robot assembly problem to build an arch structure was developed. This validation included a three part experiment set to evaluate three different factors. The first experiment takes the optimal schedule that was generated by the MIP and compares it to a hardware implementation following the optimal schedule to evaluate how well the scheduler models the assembly and to provide insight for improvement in the MIP formulation. The second experiment compares the hardware implementation of the optimal schedule against an alternative allocation policy that might be used in an in-space autonomous assembly problem where an autonomous scheduler has not been developed. Finally, the third experiment attempts a reschedule in the middle of the assembly to replicate a realistic condition where something may go wrong and the scheduler will need to be reran to determine a new set of robot to task allocations to finish the assembly.

### 3.1 Assembly Problem

The assembly project used in this work consists of taking seven blocks from a storage area, bringing them to a staging area where three subassemblies are made into two columns and the arch crossmember. Next, the three subassemblies are moved to the final assembly location where they are assembled into the finished arch. The workforce of these experiments consisted of two teleoperated robots. It should be noted that, while this system has been developed with full autonomy in mind, the scope of this experiment set is the evaluation of the optimization process. In this scope, teleoperated units are more than adequate to validate the optimization formulation. Additional, future fully autonomous experiments will be discussed in section 5.3. The following sections will fully describe the different elements in the assembly and the three experiments.

#### 3.1.1 Workspace

**FIGURE 2:**
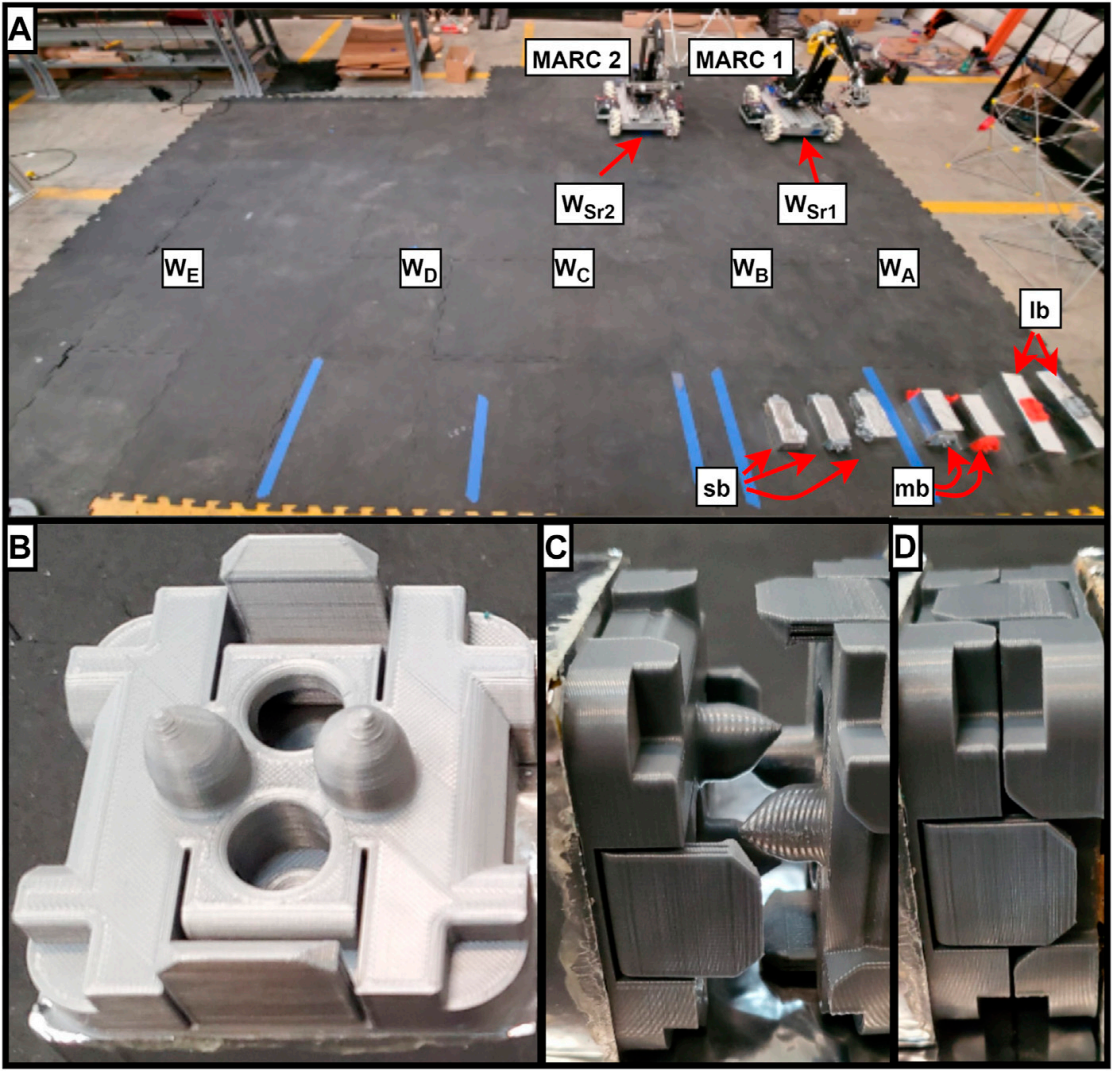
**(A)** Arch assembly workspace with location, component, and robot labels, **(B)** One side of the androgynous connector, **(C)** Both sides of the androgynous connector about to be connected, **(D)** Androgynous connector in the connected state.

The assembly workspace for this experiment took place in a 200*in* × 110*in* rectangular space. As pictured in Figure 2 panel A, there are two storage bays where the parts for the columns and horizontal crossmember are stored respectively. Both types of subassemblies (column and crossmember) have their own staging areas where they are assembled before they are brought to the final assembly area to be combined into the completed arch. In addition to these five locations, each of the robots have a starting location defined in the workspace. Table 1 provides the SAPD formulation for each of the seven reference locations.

**TABLE 1:** SAPD point definitions for the arch assembly project.

Point A (*W*_*A*_)	Point B (*W*_*B*_)	Point C (*W*_*C*_)	Point D (*W*_*D*_)
Column component storage area	Horizontal crossmember components storage area	Column component staging area	Horizontal crossmember components staging area
Properties:	Properties:	Properties:	Properties:
• Reference	• Reference	• Reference	• Reference
• (70, 185)	• (70, 155)	• (70, 115)	• (70, 85)
**Point E (*W*_*E*_)**	**Point Sr1 (*W*_*Sr*1_)**	**Point Sr2 (*W*_*Sr*2_)**	**—**
Final assembly area	MARC 1 starting location	MARC 2 starting location	**—**
Properties:	Properties:	Properties:	**—**
• Reference	• Reference	• Reference	**—**
• (70, 40)	• (20, 185)	• (20, 140)	

#### 3.1.2 Components

The components in this project consist of the seven blocks that make up the arch structure. There are two large blocks (lb1 and lb2) that will be combined with two medium blocks (mb1 and mb2) to construct the two column subassemblies. The final three blocks, two small corner blocks (sbc1 and sbc2) will combined with one small middle block (sbm1) to make the horizontal crossmember. The SAPD representation for each of these components is given in Table 2.

**TABLE 2:** SAPD component definitions for the arch assembly project.

Component lb1 (*C*_*lb*1_)	Component lb2 (*C*_*lb*2_)	Component mb1 (*C*_*mb*1_)	Component mb2 (*C*_*mb*2_)
First large block	Second large block	First medium block	Second medium block
Properties:	Properties:	Properties:	Properties:
• Large block	• Large block	• Medium block	• Medium block
• Start: *W*_*A*_	• Start: *W*_*A*_	• Start: *W*_*A*_	• Start: *W*_*A*_
State:	State:	State:	State:
• Not in position	• Not in position	• Not in position	• Not in position
• Not broken	• Not broken	• Not broken	• Not broken
• current: (70, 185)	• current: (70, 185)	• current: (70, 185)	• current: (70, 185)
**Component sbc1 (*C*_*sbc*1_)**	**Component sbc2 (*C*_*sbc*2_)**	**Component sbm1 (*C*_*sbm*1_)**	—
First small corner block	Second small corner block	Small middle block	—
Properties:	Properties:	Properties:	**—**
• Small corner block	• Small corner block	• Small middle block	**—**
• Start: *W*_*B*_	• Start: *W*_*B*_	• Start: *W*_*B*_	
State:	State:	State:	**—**
• Not in position	• Not in position	• Not in position	**—**
• Not broken	• Not broken	• Not broken	
• current: (70, 155)	• current: (70, 155)	• current: (70, 155)	

#### 3.1.3 Joints

In this assembly project, all of the joints are of a snap connector type. Pictured in Figure 2 panels B–D, these clips are androgynous and include self-aligning posts that help fine tune the alignment after a course alignment takes place. After the course alignment, force along the clip face axis will snap two clips together. There are six of these joints in the entire assembly, each of which is defined in Table 3.

**TABLE 3:** SAPD joint definitions for the arch assembly project.

Connection 1 (*K*_1_)	Connection 2 (*K*_2_)	Connection 3 (*K*_3_)
Connection between lb1 and mb1 to form the first column	Connection between lb2 and mb2 to form the second column	Connection between sbc1 and sbm1 to form part of the crossmember
Properties:	Properties:	Properties:
• Clip	• Clip	• Clip
• [*C*_*lb*1_, *C*_*mb*1_]	• [*C*_*lb*2_, *C*_*mb*2_]	• [*C*_*sbc*1_, *C*_*sbm*1_]
State:	State:	State:
• Not joined	• Not joined	• Not joined
**Connection 4 (*K*_4_)**	**Connection 5 (*K*_5_)**	**Connection 6 (*K*_6_)**
Connection between sbc2 and sbm1 to form part of the crossmember	Connection between sbc1 and mb1 to connect the crossmember to the first column	Connection between sbc2 and mb2 to connect the crossmember to the second column
Properties:	Properties:	Properties:
• Clip	• Clip	• Clip
• [*C*_*sbc*2_, *C*_*sbm*1_]	• [*C*_*sbc*1_, *C*_*mb*1_]	• [*C*_*sbc*2_, *C*_*mb*2_]
State:	State:	State:
• Not joined	• Not joined	• Not joined

#### 3.1.4 Robot Workforce

The robot workface consists of two Mobile Assembly Robot Collaborator (MARC) units designed custom in the Field and Space Experimental Robotics (FASER) laboratory. For the experimental work reported here, the teleoperators for each MARC remained constant to reduce the chance of processing time variation that might be introduced due to different skill levels or a difference in control interface familiarity. To model the processing time for different tasks, each task type was repeated thirty six times by each operator/robot to form a distribution. The expected value of each distribution was used to inform the MIP processing time and setup time information in the model. The distributions for each of these processing times will be discussed in section 4.4. The expected values, along with the rest of the robot formulation description are shown in Table 4.

**TABLE 4:** SAPD robot definitions for the arch assembly problem.

MARC 1 (*R*_*m*1_)	MARC 2 (*R*_*m*2_)
State:	State:
• Idle	• Idle
• Current: (20, 185)	• Current: (20, 140)
• Task: none	• Task: none
Properties:	Properties:
• Gripper	• Gripper
• Mobile: Yes	• Mobile: Yes
• Abilities	• Abilities
• Idle: 0.01 (s)	• Idle: 0.01 (s)
• Locomote: 6.64 (in/s)	• Locomote: 6.16 (in/s)
• Connect component: 94.33 (s)	• Connect component: 106.14 (s)
• Connect subassembly: 90.1 (s)	• Connect subassembly: 86.14 (s)
• Co-op connect component: 165.11 (s)	• Co-op connect component: 165.11 (s)
• Co-op connect subassembly: 79.58 (s)	• Co-op connect subassembly: 79.58 (s)
• Pick and Place: 41.67 (s)	• Pick & Place: 66.72 (s)
• Place: 15.61 (s)	• Place: 20.56 (s)

#### 3.1.5 Assembly Constraint Formulation

To ensure a valid assembly, components could not be assembled into subassemblies unless they were in their designated staging areas. In a similar fashion, the subassemblies could not be assembled into the final structure until they were in the final assembly area. The only robot to job restrictions were those applying to the two start jobs. These start jobs were used to insure that the robots started in the right positions. Additionally, there were no continuity constraints in this assembly problem. Four different job types were used in this assembly: idle start (*S*), move part (*M*), connect part and connect assembly (*C*). To complete these jobs, eight different operations were defined: idle, locomote, connect component, co-op connect component, connect subassembly, co-op connect subassembly, pick and place, and finally place. These operations are defined as:

• **idle:** is a very short waiting time for a robot. This is only used to initialize the robot at the correct starting location.
• **locomote:** is the motion contribution a robot can make. This is the only operation that is a velocity and is utilized anytime a robot has to move locations.
• **connect component:** is the action of picking up a component and connecting it to another component.
• **connect subassembly:** is the action of connecting both sides of the crossmember to the columns.
• **co-op connect component:** is the action of connecting one component to another with the help of a second robot.
• **co-op connect subassembly:** is the action of connecting one side of the crossmember to one column (each robot connects one of the two sides).
• **pick and place:** is the action of picking up and setting down a component. This is often used in conjunction with the locomotion velocity to describe the time it takes a robot to complete a move job type.
• **place:** is the place portion of the pick and place operation. This is used during the reschedule experiment when the damaged crossmember needs to be set down for repair.

**FIGURE 3:**
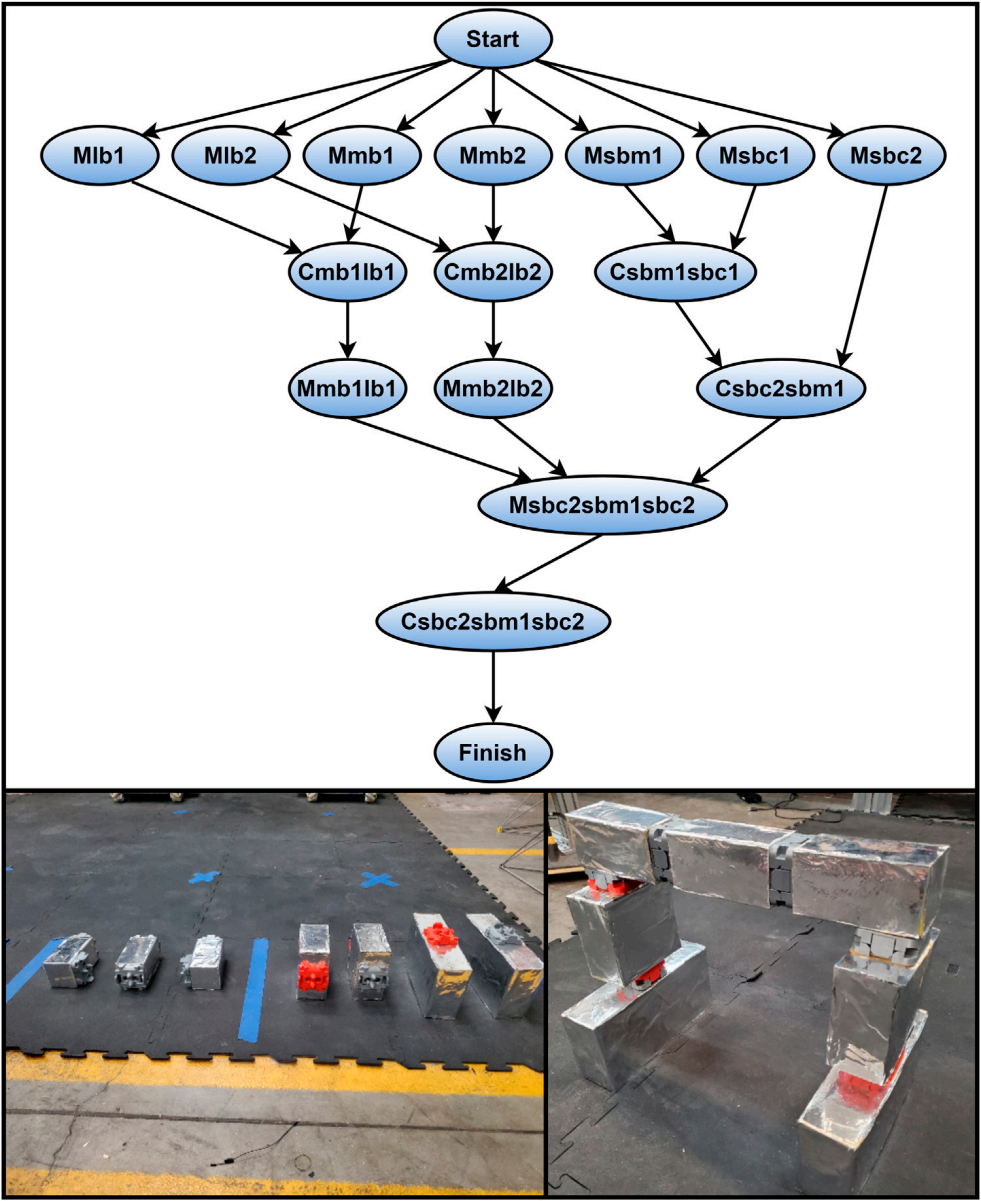
Precedence DAG for arch assembly project, Initial assembly state, Final assembly state.

The first of the four types of jobs, idle start, was a first job for each robot to insure they began in the right position. This job is analogous to the robots coming out of storage and for this experiment, it took negligible time to complete this job type. The move job type, as the name implies, was used when a component needed to be moved from one location to another. This type is unique in that the scheduler calculated the processing time for a move job as the time it took to complete the robot operation contribution (pick and place) plus the time it took the robot to traverse the distance between the starting and ending points in the job. The connect type reflected components or subassemblies being connected. This type had the sub-elements of connect component or connect subassembly. Connect component represented the time it took a robot to place and engage the clips between two components. Connect subassembly, like connect component, involved the robot connecting the clips between blocks. However, in the subassembly type, the time includes the robot having to connect both sides of the crossmember. Tables 5 and 6 provide a list and description of the different jobs that make up the assembly project and how each one could be completed, respectively. The DAG describing the precedence constraints, the initial state of the assembly, and the final state of the assembly are all shown in Figure 3.

**TABLE 5:** Jobs in the arch assembly project.

Job	Type	Component(s)	Location(s)	Description
Sr1	Start	N/A	*W*_*sr*1_	Used to ensure MARC 1 starts at the right location
Sr2	Start	N/A	*W*_*sr*2_	Used to ensure MARC 2 starts at the right location
Mlb1	Move	lb1	*W*_*A*_ → *W*_*C*_	Move the first large block from storage to staging
Mlb2	Move	lb2	*W*_*A*_ → *W*_*C*_	Move the second large block from storage to staging
Mmb1	Move	mb1	*W*_*A*_ → *W*_*C*_	Move the first medium block from storage to staging
Mmb2	Move	mb2	*W*_*A*_ → *W*_*C*_	Move the second medium block from storage to staging
Msbc1	Move	sbc1	*W*_*B*_ → *W*_*D*_	Move the first small corner block from storage to staging
Msbc2	Move	sbc2	*W*_*B*_ → *W*_*D*_	Moving the second small corner block from storage to staging
Msbm1	Move	sbm1	*W*_*B*_ → *W*_*D*_	Moving the small medium block from storage to staging
Cmb1lb1	Connect C	mb1 and lb1	*W*_*C*_	Connecting the first large and medium blocks to make the first column
Cmb2lb2	Connect C	mb2 and lb2	*W*_*C*_	Connecting the second large and medium blocks to make the second column
Mmb1lb1	Move	mb1 and lb1	*W*_*C*_ → *W*_*E*_	Move the first column from staging to the final assembly location
Mmb2lb2	Move	mb2 and lb2	*W*_*C*_ → *W*_*E*_	Move the second column from staging to the final assembly location
Csbm1sbc1	Connect C	smb1 and sbc1	*W*_*D*_	Connecting the small middle block with the first small corner block to make part of the horizontal crossmember
Csbc2sbm1	Connect C	sbc2 and sbm1	*W*_*D*_	Connecting the small middle block with the second small corner block to make part of the horizontal crossmember
Msbc1sbm1sbc2	Move	sbc1 and sbm1 and sbc2	*W*_*D*_ → *W*_*E*_	Move the crossmember to the final assembly location
Csbc1sbm1sbc2	Connect S	sbc1 and sbm2 and sbc2	*W*_*E*_	Connect both sides of the crossmember to the two columns

**TABLE 6:** Process plans and operations for each of the types of jobs.

Job type	Process plan(s)	Operation(s)
Start	p0	(Idle)
Move	p0	(Pick and Place)
Connect C	p0	(Connect component)
	p1	(Co-op connect component, Co-op connect component)
Connect S	p0	(Connect subassembly)
	p1	(Co-op connect subassembly, Co-op connect subassembly)

### 3.2 Experiment Implementation

**FIGURE 4:**
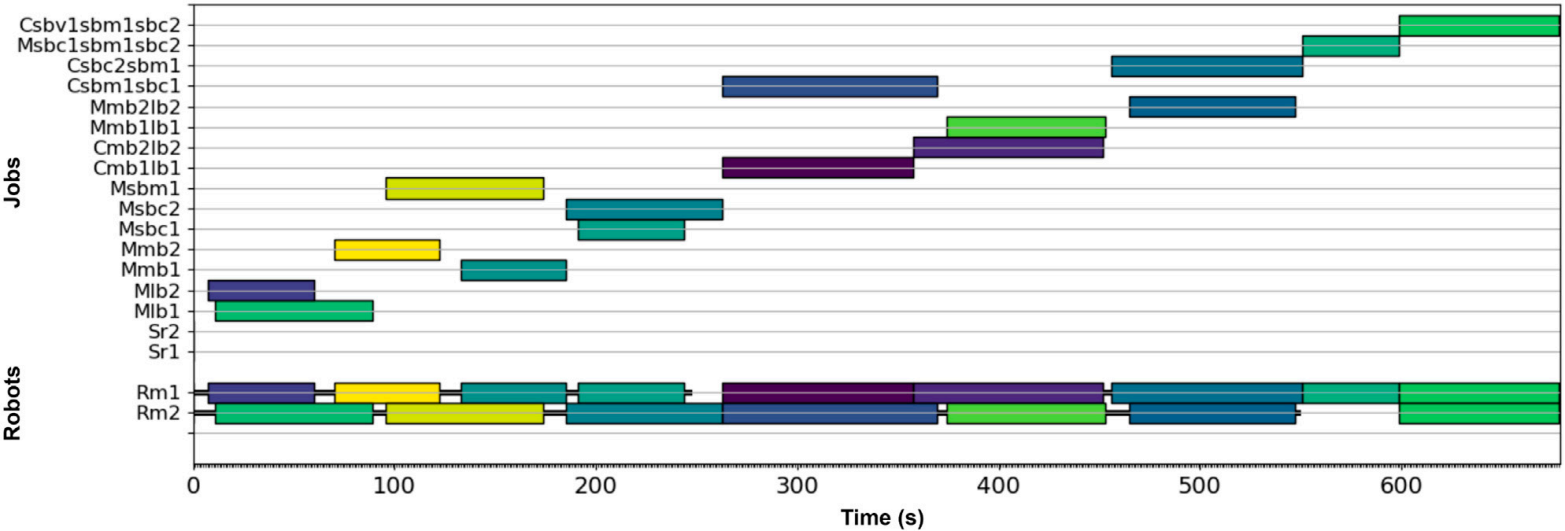
Optimal schedule generated by the mixed integer program. The bottom portion of the graph shows the robot to task allocation and the upper portion of the graph shows the task sequencing. The job times between the two portions are color coded and spatially synced.

As noted above, the processing time information for each of the operations in the assembly project was generated through thirty six instances of each robot completing each type of operation. Each set of thirty six contained slight variations likely to be seen in the actual assembly project. For example, to evaluate the connect component processing time, combinations of the three different block sizes (small, medium, and large) and three different spacings between pre-connected blocks were used. This experimentally generating the processing times to inform the SAPD, providing the stochastic variation information present due to operator control and slight changes present due to different grips required for the three block types to be modeled. Each robot was controlled by the same operator through all of the experiments to preserve the distributions acquired for the SAPD. The MIP scheduler was implemented using the Gurobi Python API (Gurobi Optimization, 2021) on a Windows 10 computer with 16 GB of RAM and a 2.20 GHz i7 CPU. The solution generated by the MIP was used as the optimal schedule in all three experiments. The optimal schedule was solved for in 9.58 s. Figure 4 provides a picture of the optimal schedule. The bottom two rows in the graph show which robot worked one what job at what time in the assembly project. The black lines between jobs show when a robot would need to travel between jobs and the blank gaps represent when a robot is waiting to start its next task. The upper portion of the graph shows when each job was processed in the assembly project. The jobs times in the upper and lower portions of the graph are color synced to clarify task allocation when two robots are working at the same time. The projected completion time for the assembly was 679 s.

#### 3.2.1 Full Assembly

The full assembly experiment consisted of two complete runs of the assembly project following the task sequence and allocation in the optimal schedule. During the assembly runs, there were a few instances of unexpected errors. In a deployed scenario, these errors would lead to a reschedule and reallocation of the robots. Since the third experiment tests this ability, the other experiments focused on other evaluations. For the first two experiments, if an unexpected error occurred, such as a battery running low or a connection clip breaking, all of the operators were immediately notified and the experiment was paused while the issue was resolved. Once resolved, everything continued from the point where the error occurred, allowing for the down time due to the error to be factored out of the processing times making the comparison between the schedule and the hardware implementation valid. It should be noted that failures in the experiment, such as a gripper needing to be readjusted or a second attempt at making a clip connect, were left in the experiment since they are realistic elements in the assembly problem.

#### 3.2.2 Alternative Policy Task Allocation

To evaluate how the completion time of a hardware implementation of the optimal schedule compares to an assignment method that did not have the task sequencing and allocation analysis capability, an alternative assignment policy was chosen that reflects a realistic strategy to complete a new assembly project. This alternative policy allocated tasks based on their type to a specific robot. MARC 1 was faster at completing the connect component operations than MARC 2 so all of the connection type jobs were given to MARC 1. Similarly, MARC 2 was given all of the move job types. To give this policy the best chance against the optimal policy, the jobs were completed in a way that would minimized the amount of time one robot was waiting on the other. While this changed the task sequencing slightly in the alternative policy hardware implementation, it allowed for the best comparison between the optimal task allocation and the alternative policy allocation.

#### 3.2.3 Replanning Error Correction

To test the ability of this scheduler to reschedule, one of the motivations in the development of the task assignment framework, a failure was forced near the end of the assembly. Once the crossmember arrived at the assembly location, the end of the crossmember was removed from the other two components. This required a reassignment with a new starting configuration reflecting the current state of the assembly. Jobs were inserted to set the crossmember down, repair it, and then finish the assembly.

## 4 Results

The following sections will report the results from the experiments. Due to the teleoperated nature of the experiments, the hardware runs were video recorded and the processing times were pulled from the timestamps in the video. For each robot working on each job, the timestamp was recorded for when a robot started and finished traveling between jobs and when it started and completed working on each job. The following sections report the results from the three experiments.

### 4.1 Full Assembly

Two full assembly runs following the optimal schedule were completed. In the first, part way through the assembly, one of the connection clips broke requiring the experiment to be paused and the component replaced. In the second assembly, an electronics issue occurred also requiring the experiment to be paused. The presence of these errors further highlight the necessity of a system capable of articulating the states needed for repair and reallocating robots autonomously. As described above, both of these pauses were removed from the data in post-processing and the resulting data was verified against the footage.

#### 4.1.1 First Full Assembly Run

**FIGURE 5:**
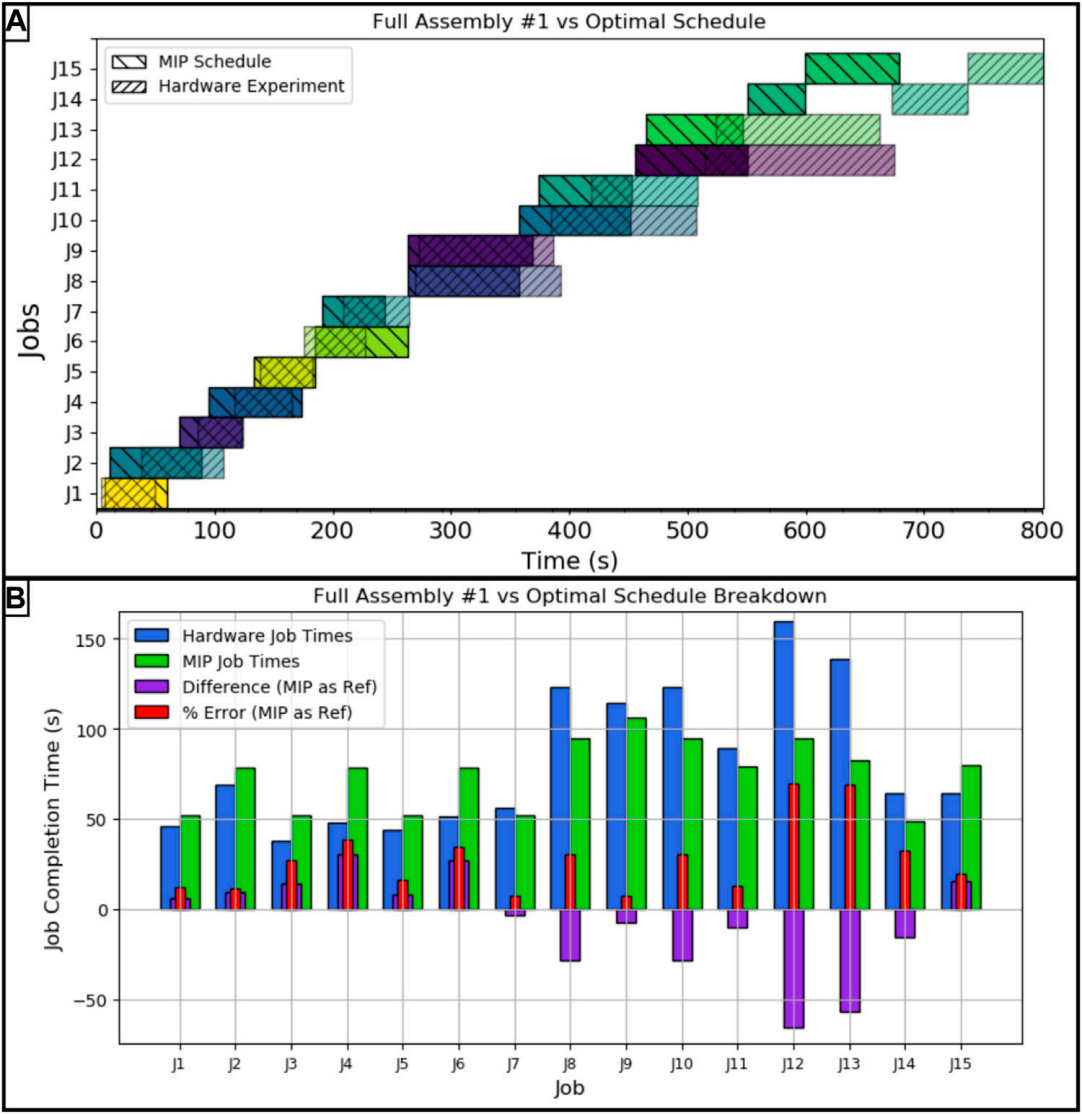
**(A)** Hardware vs. MIP optimal schedule for the first full assembly run, **(B)**: Job by job breakdown with error for the first full assembly run. The job key is as follows: *J*1: *Mlb*2, *J*2: *Mlb*1, *J*3: *Mmb*2, *J*4: *Msbm*1, *J*5: *Mmb*1, *J*6: *Msbc*2, *J*7: *Msbc*1, *J*8: *Cmb*1*lb*1, *J*9: *Csbm*1*sbc*1, *J*10: *Cmb*2*lb*2, *J*11: *Mmb*1*lb*1, *J*12: *Csbc*2*sbm*1, *J*13: *Mmb*2*lb*2, *J*14: *Msbc*1*sbm*1*sbc*2, *J*15: *Csbc*1*sbm*1*sbc*2.

In Figure 5 panel A the comparison between the first full assembly run and the optimal MIP schedule is shown. This assembly took 801 s to complete, an 18.14% error in overall completion time of the project. A job by job break down of the differences and percent errors between the optimal projected schedule and this hardware trial is shown in Figure 5 panel B. The largest two errors occurred when dealing with the horizontal crossmember. As shown in the same figure and panel, all of the jobs moving components from the storage to the staging area took a few second less than the predicted time, on average. Alternatively, all of the jobs dealing with the subassemblies took longer than predicted aside from the final job where the two robots connected the horizontal crossmember to the two columns.

#### 4.1.2 Second Full Assembly Run

**FIGURE 6:**
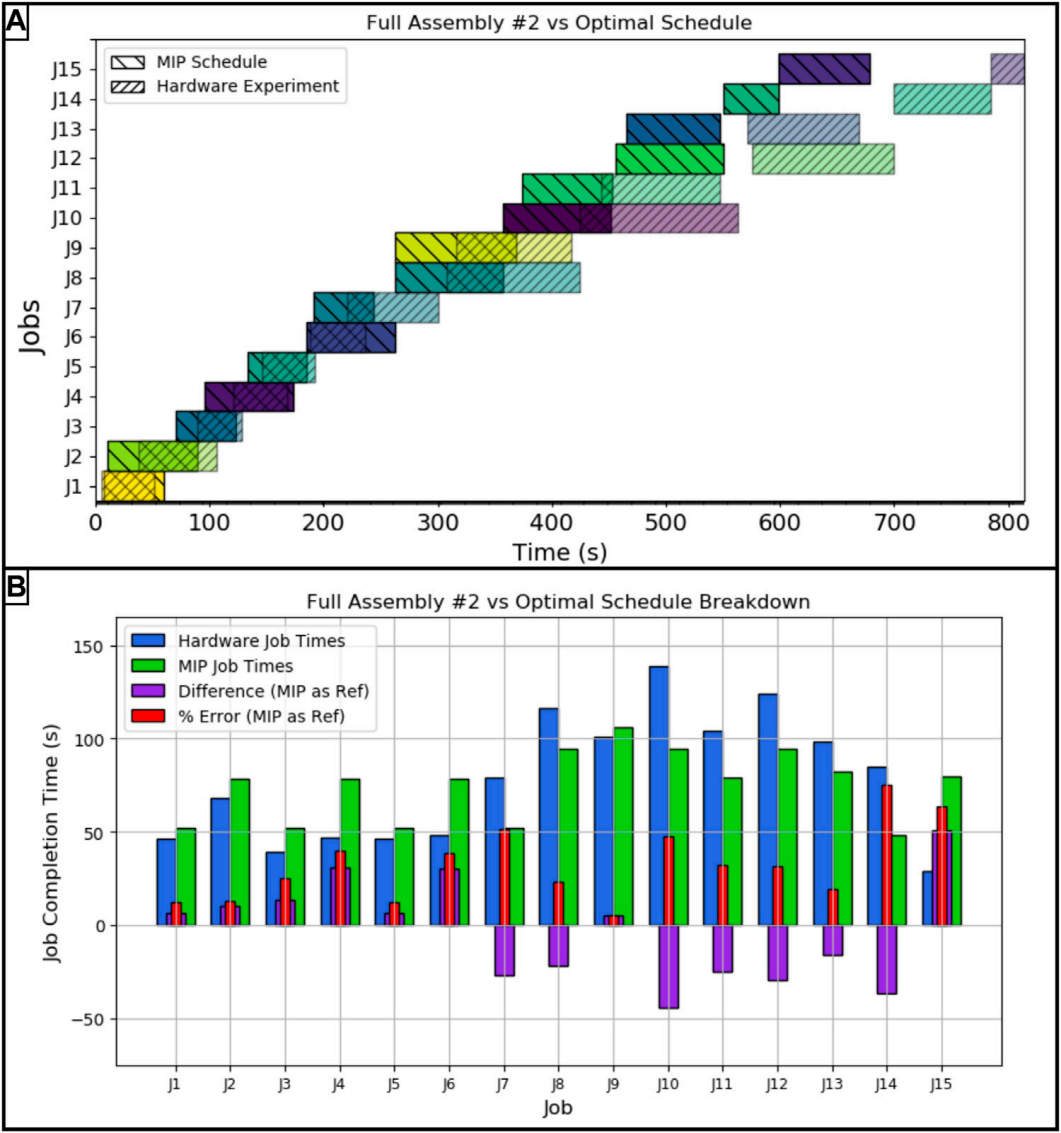
**(A)** Hardware vs MIP optimal schedule for the second full assembly run, **(B)** Job by job breakdown with error for the second full assembly run. The job key is as follows: *J*1: *Mlb*2, *J*2: *Mlb*1, *J*3: *Mmb*2, *J*4: *Msbm*1, *J*5: *Mmb*1, *J*6: *Msbc*2, *J*7: *Msbc*1, *J*8: *Cmb*1*lb*1, *J*9: *Csbm*1*sbc*1, *J*10: *Cmb*2*lb*2, *J*11: *Mmb*1*lb*1, *J*12: *Csbc*2*sbm*1, *J*13: *Mmb*2*lb*2, *J*14: *Msbc*1*sbm*1*sbc*2, *J*15: *Csbc*1*sbm*1*sbc*2.

Similar to the first full assembly run, the second assembly took 814 s to complete, a 20.06*%* error from the optimal schedule. Like the first assembly, the schedule comparison shown in Figure 6 shows similar error trends where the moving of parts between the storage and staging areas were slightly faster than predicted and the jobs dealing with the subassemblies took longer than projected. In the second assembly run, the largest difference between actual and projected comes from moving the crossmember into place.

### 4.2 Alternative Policy

**FIGURE 7:**
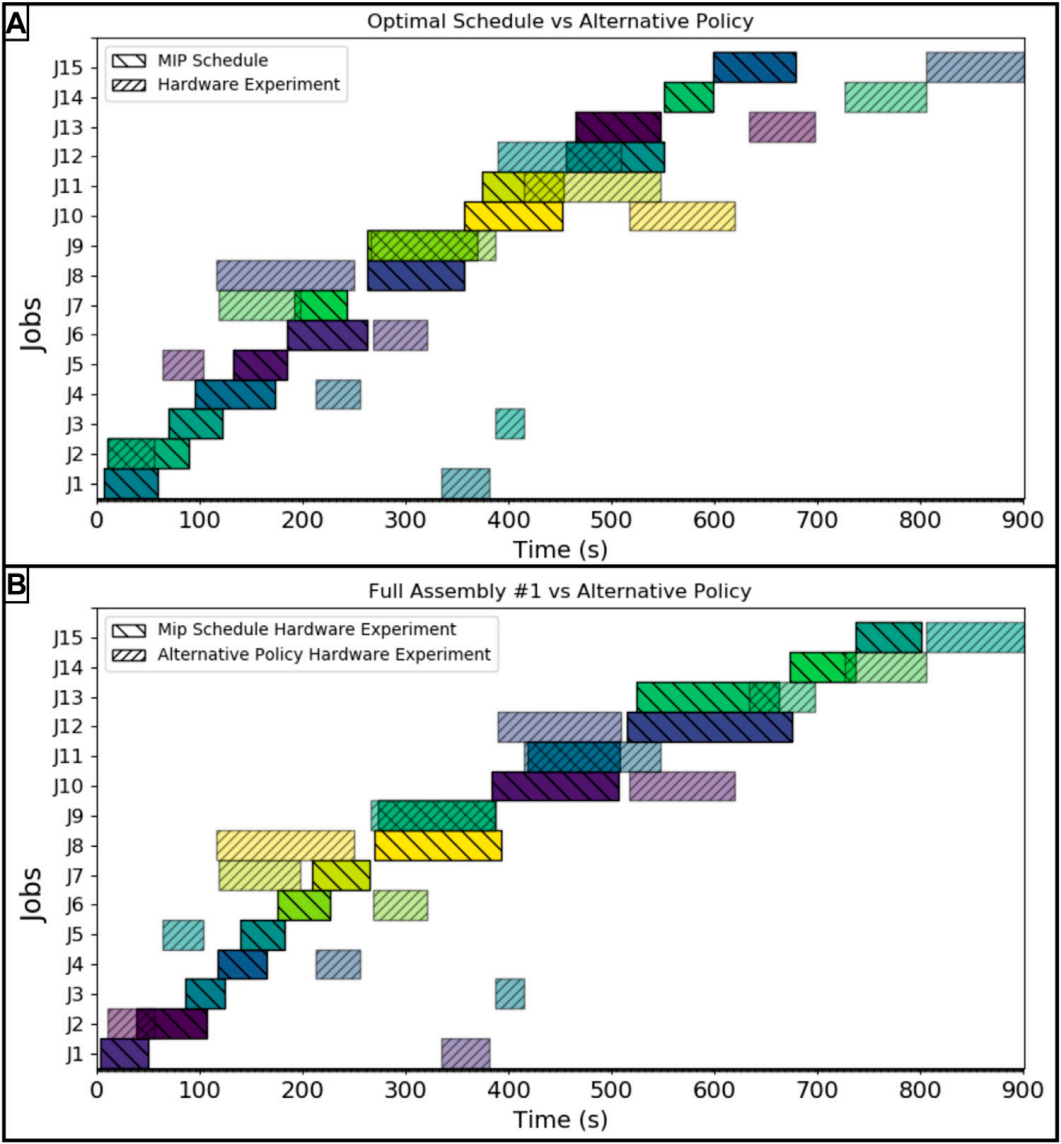
**(A)** Hardware experiment following alternative policy compared against the optimal MIP scheduled, **(B)** Hardware experiment following alternative policy compared against hardware experiment following the optimal schedule. The job key is as follows: *J*1: *Mlb*2, *J*2: *Mlb*1, *J*3: *Mmb*2, *J*4: *Msbm*1, *J*5: *Mmb*1, *J*6: *Msbc*2, *J*7: *Msbc*1, *J*8: *Cmb*1*lb*1, *J*9: *Csbm*1*sbc*1, *J*10: *Cmb*2*lb*2, *J*11: *Mmb*1*lb*1, *J*12: *Csbc*2*sbm*1, *J*13: *Mmb*2*lb*2, *J*14: *Msbc*1*sbm*1*sbc*2, *J*15: *Csbc*1*sbm*1*sbc*2.

As stated in section 3, the alternative assignment policy where MARC 1 completed only the connecting type jobs and MARC 2 completed all of the move type jobs, was used to evaluate the benefit of using the optimal schedule against an alternative, realistic assignment policy in an autonomous scenario. The job completion time results for this hardware trial are compared against the optimal schedule and the first hardware implementation of the optimal schedule in Figure 7 panel A and panel B respectively. Even with the reordering of task sequencing to complete the alternative policy in the optimal way, the alternative policy makespan was 901 s, yielding an error of 32.89% from the optimal schedule projection and a 12.48% error longer from the first hardware run implementing the optimal schedule.

### 4.3 Replanning

Using the same computer that generated the original schedule, the reschedule was solved in 0.066 s (negligible) time and the assembly proceeded with MARC 1 being retasked (since it was the closest to the damaged part) to set the partial crossmember down, repair the broken joint, and finish the assembly. After the repair portion of the jobs, the new schedule matched the original task allocation, completing final job with both MARCs to attach the crossmember to the two columns.

### 4.4 Processing Times

**FIGURE 8:**
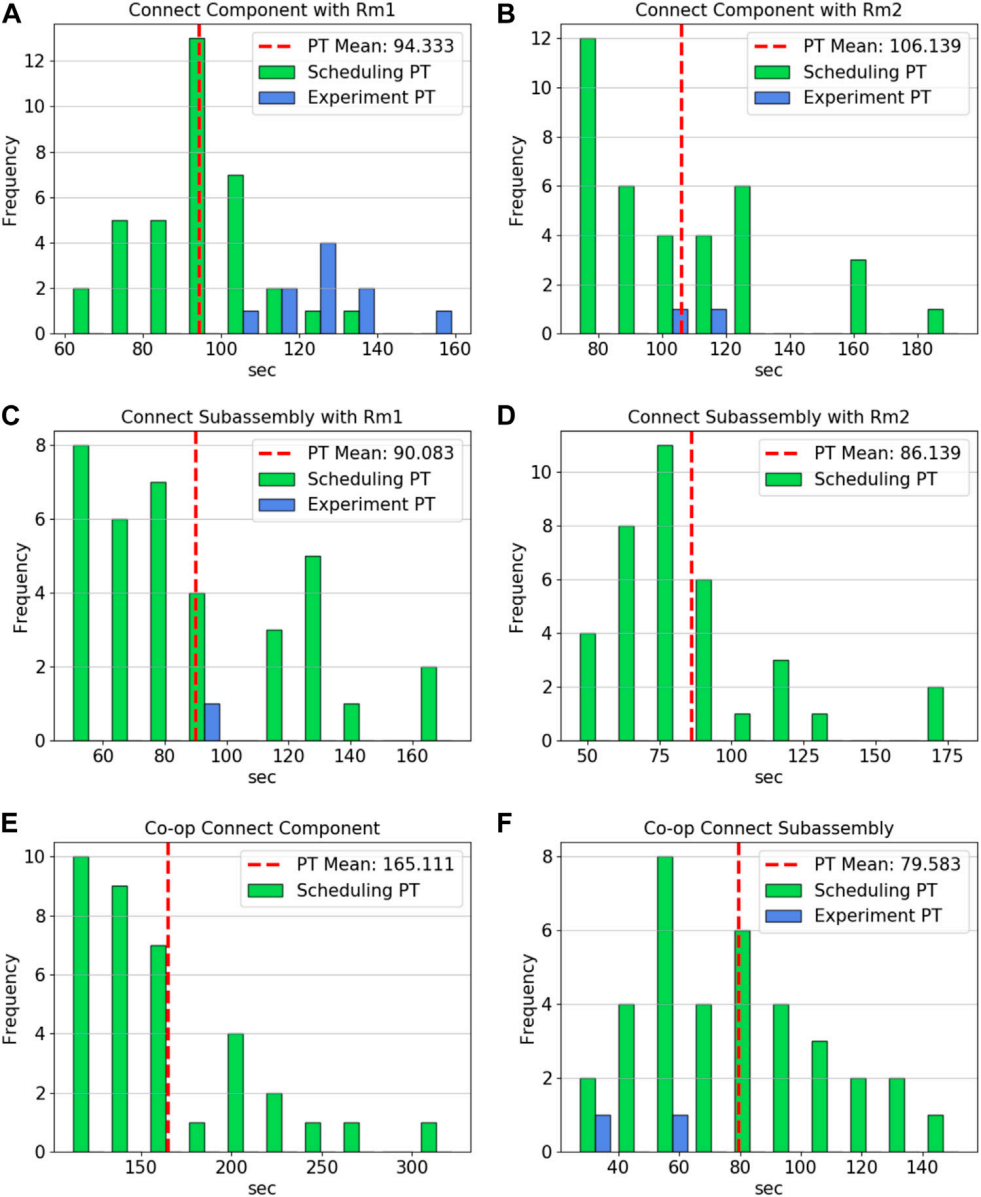
**(A)** MARC 1 connect component processing times for scheduler and hardware instances, **(B)** MARC 2 connect component processing times for scheduler and hardware instances, **(C)** MARC 1 connect subassembly processing times for scheduler and hardware instances, **(D)** MARC 2 connect subassembly processing times for scheduler (there were no hardware instances), **(E)** Co-op connect component processing times for scheduler (there were no hardware instances), **(F)** Co-op connect subassembly processing times for scheduler and hardware instances.

**FIGURE 9:**
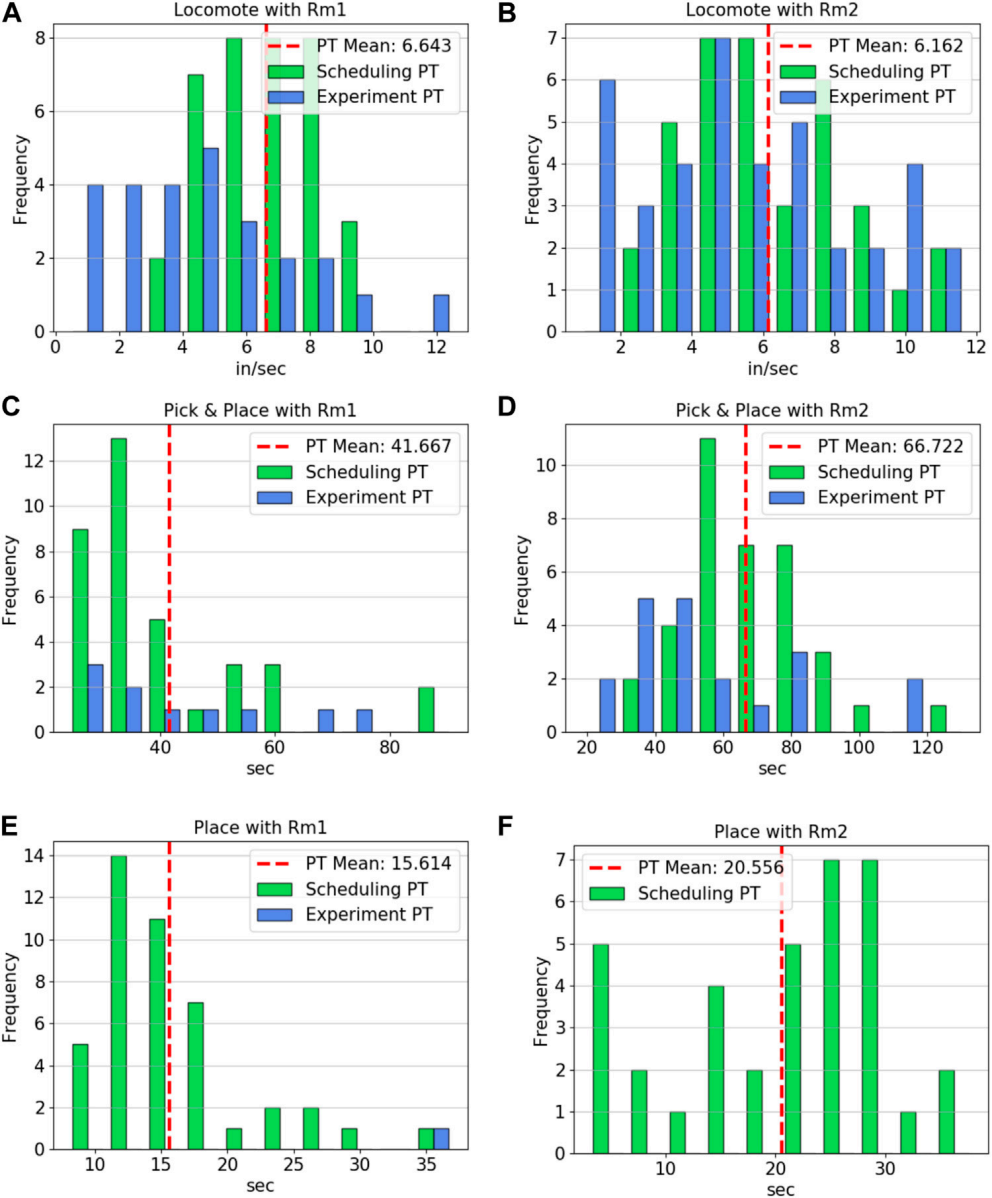
**(A)** MARC 1 locomote velocity for scheduler and hardware instances, **(B)** MARC 2 locomote velocity for scheduler and hardware instances, **(C)** MARC 1 pick and place processing times for scheduler and hardware instances, **(D)** MARC 2 pick and place processing times for scheduler and hardware instances, **(E)** MARC 1 place processing times for scheduler and hardware instances, **(F)** MARC 2 place processing times for scheduler (there were no hardware instances).

When applicable, the processing times measured during the hardware experiments were plotted over the distributions of the data used to generate the expected values in the scheduler’s processing time entries. Figure 8 contains the processing time distributions for the connect component, connect subassemblies, co-op connect component, and co-op connect subassemblies operations for both of the robots. The hardware processing times for MARC 1’s connect component appear to be in the upper range of those seen in the data taken for ability property. As shown in panel A, it took about 35 seconds longer, on average, than the value used in the scheduler. This corresponds to the higher error in the connect component job types shown in the schedule. In contrast, MARC 2, which only had a few instances of connecting a component throughout the hardware trials, had a processing time very close to the expected value of the input data when completing *Csbm*1*sbc*1. Panels C and D show only one and no hardware data points for MARC 1 and 2, respectively, since the optimal scheduler used both robots to connect the subassemblies together. The one entry for MARC 1 reflects the alternative policy requiring MARC 1 to handle all of the connection job types on its own. Similarly, the only co-op operation utilized was connecting the subassembly shown in panel F. The final three operations, locomote, pick and place, and place, show similar results in Figure 9. The locomote instances for both robots, shown in panels A and B, are similar to those used by the scheduler. The hardware processing times have not been corrected for the extra distance the robots had to travel to avoid colliding (since path planning was not part of this experiment set). The similar distributions between the hardware trials and abilities data indicates that this was not a large contributor to the error seen between the optimal schedule and the results. The pick and place operations, panels C and D, show that the distribution ranges are about the same but with instances 20–30 s faster and slower than the expected value of the input data.

## 5 Discussion

The developed SAPD was able to successfully articulate all of the elements present in the assembly problem that were important to task assignment and successfully encode the constraints in a way that could be passed to a schedule generator to generate task sequences and allocations. The MIP schedule generator was then able to take these elements and constraints to solve for an optimal schedule which proved to be more optimal to follow than an alternative, less informed, viable scheduling policy. The below sections will discuss advantages and limitations present in the formulations and will end with future work suggestions.

### 5.1 Advantages

The SAPD is capable of describing the elements present in an assembly problem. It has a flexible formulation that allows to adapt between simple, small scale, assembly projects, up to large, stochastic projects. The ability to record both state and property information allows it contain information that may be needed by other elements of an autonomy suite used in in-space assembly scenarios. The MIP formulation proved to be capable of modeling distance considerations, continuity constraints, and the other core constraint requirements to ensure a valid assembly schedule. By the nature of the branch and bound solving method, used for MIP formulations, it was able to guarantee an optimal schedule based on the input data and contained the ability to give a measure of how far from the optimal solution the generated schedule might be. At the experimental scale presented in this work, schedule generation was very fast, making it usable for rescheduling, an important consideration for in-space applications where the delay or lack of communication can make teleoperated rescheduling impractical or impossible.

### 5.2 Limitations

Using the MDP formulation in the SAPD may reach intractable state numbers for very large assemblies. While this is not a direct limitation to the formulation, additional work may need to be done to determine the best way to approximate the states or downsample to the a smaller, important state set. This is additional work that would need to feed the problem definition, adding another capability requirement in the overall autonomy system. The MIP formulation, in its current form, does not take into account the spatial footprint of the robots. While it did not greatly impact the overall assembly runs above, it did cause some of the robot interactions to go unaccounted for in the model. One possible mitigation is to factor in path routing in the MIP. This, however, leads to the second limitation. The MIP treats the whole assembly problem all at once, instead of solving it temporally from beginning to end. If a change is made, the overall assembly changes and the solving process needs to start over. While this can be mitigated by feeding in the previous solution to “warm start” the solver, it could cause limitations if the solving time is too large. This is a potential concern for large projects since the addition of jobs or robots will increase the problem space significantly thereby increasing the computation time required to produce a schedule. Future experiments need to be done to determine the scale limitations of the MIP formulation.

### 5.3 Future Work

Future work for the SAPD will include additional scenarios with variations in assembly constraints to provide an extended evaluation into the flexibility of the formulation to describe the wide range of assembly projects present in the in-space assembly domain. Ongoing work is being done to generate experiments that will have similar features to building support structures for Martian or Lunar bases. Future work for the MIP formulation will include constraints that allow additional spatial consideration. One possible way to do this is to insert required delays between start times when robots are set to occupy the same location based on the expected completion time between the incoming and outgoing units. This will need to be paired with a consideration for when two robots are working on the same job at the same location. Another addition to the MIP could be to map locations to the operations rather than just the jobs. This will allow the formulation to handle instances where two robots need to collaborate on a job from locations that are spatially separate. Additionally, alternative methods to generate schedules should be explored, including a stochastic mixed integer program, to better incorporate the stochastic distributions from the SAPD into the schedule generation. An interesting hybrid approach might be to use the MIP formulation to generate an initial optimal schedule paired with a faster, less optimal algorithm to modify the schedule to fix issues that may arise during the assembly. Finally, making the teleoperated robots autonomous and running additional assembly experiments will allow for a clearer analysis of how the model handles the stochastic characteristics present in the hardware systems and state estimation algorithms in an autonomous robotic system.

### 5.4 Conclusion

A key element in the expansion of space exploration is the use of autonomous robots to build and maintain structures for the arrival of humans or to handle missions humans can not. To accomplish this in a realistic, stochastic environment, one of the necessary elements of autonomy is the ability to generate good task sequences and task allocations. The novel work presented here developed a formulation capable of articulating the necessary elements present in an autonomous assembly problem and a framing that is capable of including state transitions during a mid-assembly repair. Additionally, this work developed a formulation to generate optimal schedules and provided a preliminary validation of the developed methodology using teleoperated hardware assembly experiments to build a structure and reallocate mid-assembly to repair a broken component. The continued development of this work has promise to provide an important piece of the puzzle in using autonomous robots in the overall quest of space exploration and colonization.

## Data Availability

The raw data supporting the conclusion of this article will be made available by the authors, without undue reservation.

## Abbreviations

DAG, directed acyclic graph; FCG, fully connected graph; ISAAP, in-space autonomous assembly problem; MDP, Markov decision process; MIP, mixed integer program; SAPD, stochastic assembly problem definition.
